# Universal health coverage provisions for women, children and adolescents

**DOI:** 10.2471/BLT.19.249474

**Published:** 2020-02-01

**Authors:** Elizabeth Mason, Gita Sen, Alicia Ely Yamin, on behalf of the United Nations Secretary-General’s Independent Accountability Panel for Every Woman, Every Child, Every Adolescent

**Affiliations:** a Institute for Global Health, University College London, Gower Street, London, WC1E 6BT, England.; b Ramalingaswami Centre on Equity & Social Determinants of Health, Public Health Foundation of India, Bangalore, India.; c Petrie-Flom Center for Health Law Policy and Bioethics at Harvard Law School, Harvard TH Chan School of Public Health, Cambridge, United States of America.

The United Nations Secretary-General’s Independent Accountability Panel for Every Woman, Every Child, Every Adolescent considers universal health coverage (UHC) a political investment and implementation opportunity to advance the health of women, children and adolescents. However, UHC cannot be universal unless everyone is reached, including those in fragile settings.^1^^,^^2^

The United Nations General Assembly Political Declaration of the High-Level Meeting on UHC^3^ highlighted the central role of primary health care, which comprises community engagement, primary care and multisectoral action.^4^ Primary health care can meet most health needs throughout life through quality, community-based preventive and promotive health services, with referrals when necessary. All countries have an obligation under international law to deliver these services in ways that respect, protect and fulfil human rights, uphold dignity and ensure financial protection; governments must be held accountable for doing so.^5^ Legal accountability based on respect for these rights is needed to prevent and remedy health inequities and the structural injustices that underlie them. For instance, a recent study across Ghana, Guinea, Myanmar and Nigeria revealed that around one third of women in health facilities experience mistreatment and abuse, particularly around childbirth.^6^ The study highlighted the need to understand drivers and structural dimensions of human rights violations, including gender-based inequalities, discrimination and violence.^6^

Child survival, adolescent development and women’s health gains are at risk^7^ unless UHC efforts focus on essential health services, quality of care, access to public health goods and on ensuring that legal determinants of health are in place, including for sexual and reproductive health, and rights.^8^^-^^10^

The panel is committed to promoting UHC accountability to ensure that all women, children and adolescents can access the quality services they need without financial hardship, allowing them to realize their rights to health and wellbeing. 

To achieve this goal, governments need to include essential health services for women, children and adolescents throughout the life course in their national UHC packages. Countries should implement the World Health Organization’s guidance on UHC and primary health care governance, financing, health workforce, equity and quality of care, among others.^11^

Multisectoral engagement should be enabled through legal and policy frameworks, including to address rights violations related to discrimination in health care, restricted sexual and reproductive health and rights, and gender-based violence. Governments should ensure nutrition for women, children and adolescents, and address gendered barriers to water and sanitation, and environmental health. 

Through courts, parliamentarians and civil society, governments should support inclusive social, political and legal accountability and meaningful oversight to achieve health and sustainable development goals and human rights. They should ensure legal protections for civic space, freedoms of information and association. Social accountability and feedback from diverse groups, including women, children and adolescents on whether UHC is meeting their needs is also important.^8^

Governments, academia and development partners should collaborate to collect and analyse data across all sectors. Standardized data-collection systems are needed to identify and address data gaps and biases.

Governments and partners, especially civil society, should use rights-, gender- and equity-based approaches to identify patterns of discrimination and marginalization within the health system and beyond resulting from structural injustices based on power, social, economic and political differentials. Appropriate tools should be deployed to analyse gaps, promote equity and reach women, children and adolescents in fragile settings.^12^

The international community needs to address transnational factors that limit countries’ capacities to deliver UHC, such as constraints around prioritization, fiscal space and financing, pricing and production of products, inequitable access to global public health goods, insufficient support for fragile states and migrants’ health, among other concerns.

Governments and development partners should commit publicly to these actions and to being held accountable for delivering UHC and primary health care in a way that respects, protects and fulfils human rights. All societies and individuals would benefit from these actions because they contribute to health and well-being and to sustainable development, equity and security.^10^
